# Structure and mechanism of TagA, a novel membrane-associated glycosyltransferase that produces wall teichoic acids in pathogenic bacteria

**DOI:** 10.1371/journal.ppat.1007723

**Published:** 2019-04-19

**Authors:** Michele D. Kattke, Jason E. Gosschalk, Orlando E. Martinez, Garima Kumar, Robert T. Gale, Duilio Cascio, Michael R. Sawaya, Martin Philips, Eric D. Brown, Robert T. Clubb

**Affiliations:** 1 Department of Chemistry and Biochemistry, University of California, Los Angeles, Los Angeles, United States of America; 2 UCLA-DOE Institute of Genomics and Proteomics, University of California, Los Angeles, Los Angeles, United States of America; 3 Department of Chemistry and Biomedical Sciences, McMaster University, Hamilton, Ontario, Canada; 4 Molecular Biology Institute, University of California, Los Angeles, Los Angeles, United States of America; National Jewish Health, UNITED STATES

## Abstract

*Staphylococcus aureus* and other bacterial pathogens affix wall teichoic acids (WTAs) to their surface. These highly abundant anionic glycopolymers have critical functions in bacterial physiology and their susceptibility to β-lactam antibiotics. The membrane-associated TagA glycosyltransferase (GT) catalyzes the first-committed step in WTA biosynthesis and is a founding member of the WecB/TagA/CpsF GT family, more than 6,000 enzymes that synthesize a range of extracellular polysaccharides through a poorly understood mechanism. Crystal structures of TagA from *T*. *italicus* in its apo- and UDP-bound states reveal a novel GT fold, and coupled with biochemical and cellular data define the mechanism of catalysis. We propose that enzyme activity is regulated by interactions with the bilayer, which trigger a structural change that facilitates proper active site formation and recognition of the enzyme’s lipid-linked substrate. These findings inform upon the molecular basis of WecB/TagA/CpsF activity and could guide the development of new anti-microbial drugs.

## Author summary

Gram-positive bacteria cause thousands of deaths in the United States each year and are a growing health concern because many bacterial strains have become resistant to commonly used antibiotics. One of the most abundant polymers displayed on the surface of Gram-positive bacteria is wall teichoic acid (WTA), a negatively charged carbohydrate polymer that has critical functions in cell division, morphology, adhesion and pathogenesis. The WTA biosynthetic pathway has drawn significant interest as a drug target because clinically important methicillin-resistant *S*. *aureus* (MRSA) strains that lack WTA are defective in host colonization and re-sensitized to β-lactam antibiotics. To understand how bacteria produce WTA, we determined the structure and deduced the enzymatic mechanism of TagA, an important enzyme that is required for WTA synthesis. This research reveals a new method for enzyme regulation, whereby peripheral membrane association enables TagA to adopt its active form as a monomer. As TagA enzymes are highly conserved in bacteria, they can be expected to operate through a similar mechanism. The results of this work provide insight into WTA biosynthesis and could lead to innovative approaches to treat infections caused by pathogenic bacteria.

## Introduction

The thick peptidoglycan (PG) sacculus that surrounds Gram-positive bacteria maintains cellular integrity and is affixed with proteins and glycopolymers that have important roles in microbial physiology and host-pathogen interactions. In *Staphylococcus aureus* and other Gram-positive bacteria, wall teichoic acids (WTAs) are a major component of the cell wall, constituting up to 60% of its dry mass [1]. WTAs have essential functions, including regulating PG biosynthesis, morphogenesis, autolysin activity, immune evasion, resistance to host cationic antimicrobial peptides, and pathogenesis [2–9]. The WTA biosynthetic pathway has drawn considerable interest as an antibiotic target, as genetically eliminating WTA production in clinically important Methicillin-resistant *Staphylococcus aureus* (MRSA) re-sensitizes it to β-lactam antibiotics and attenuates its virulence [2, 3].

**Fig 1:**
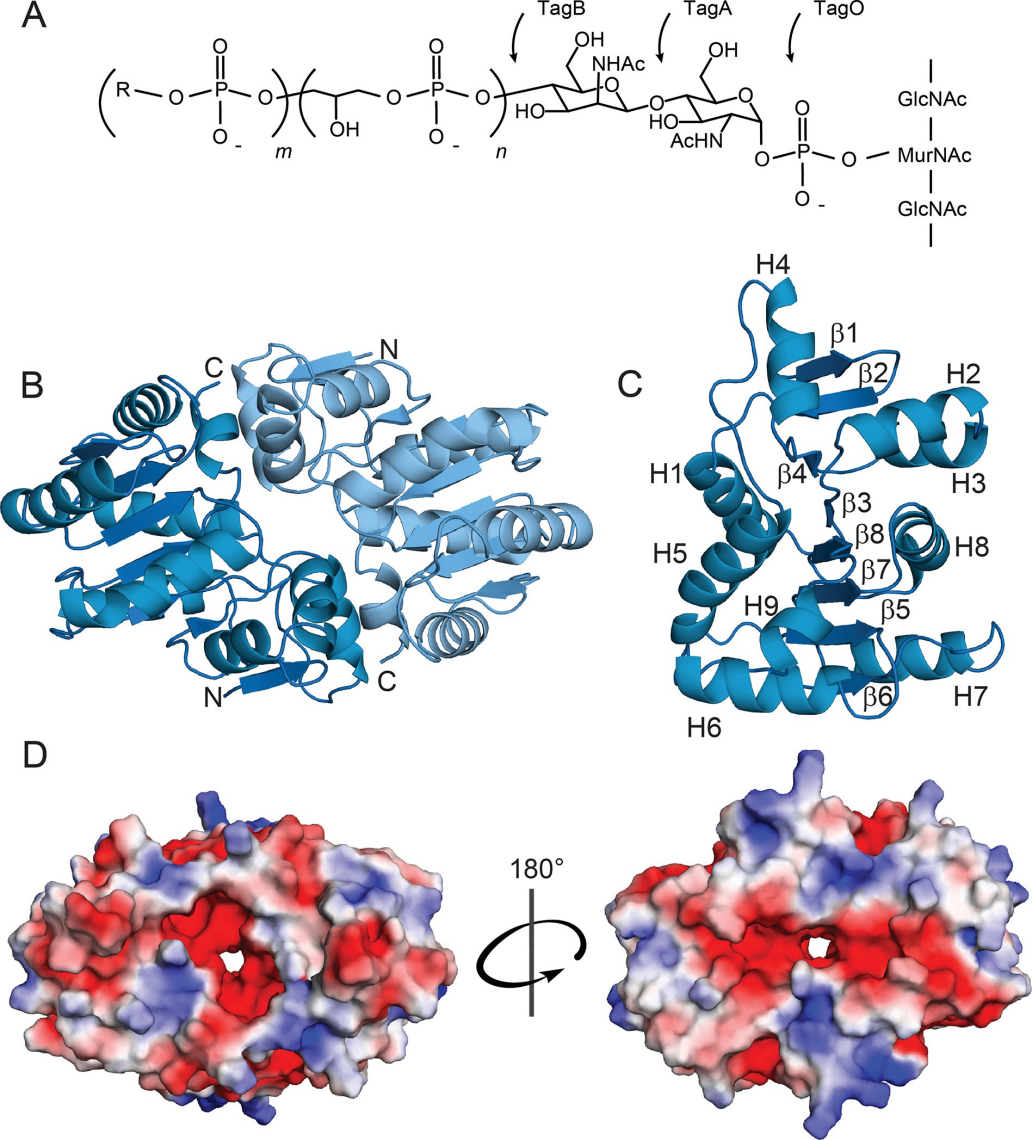
The WTA linkage unit and TagA structural characteristics. (A) WTA linkage unit and polymer. The linkage unit is attached to the peptidoglycan via the C6-hydroxyl of *N*-acetylmuramic acid and is composed of a GlcNAc (TagO-catalyzed), ManNAc (TagA-catalyzed), and *n =* 2–3 glycerol phosphates (TagB- and TagF-catalyzed). R = glycerol or ribitol, *m =* 40–60. (B) Cartoon ribbon representation of TagA^ΔC^ from *T*. *italicus*. Apo-TagA^ΔC^ crystallizes as a dimer. The dimer interface is over 1000 Å in surface area and is formed by buried hydrophobic residues. (C) TagA^ΔC^ protomer with secondary structural elements indicated. H = alpha helix, β = beta strand. (D) Electrostatic surface representation of the TagA^ΔC^ dimer. Negatively charged (*red*), neutral (*white*), and positively charged (*blue*) residues are indicated. Rotation of 180° about the dimer interface allows visualization of the pore.

WTA polymers are constructed from polymerized alditol-phosphate subunits that are attached to the cell wall via a disaccharide-containing linkage unit. While the chemical structure of the main chain of the polymer can vary, the structure of the linkage unit is highly conserved across different species of Gram-positive bacteria and is composed of an *N-*acetylmannosamine (ManNAc) (β1→4) *N*-acetylglucosamine (GlcNAc) disaccharide appended to one to three glycerol-3-phosphate (GroP) groups [10] (**Fig 1A**). The linkage unit performs a key function in WTA display, connecting the WTA polymer to the C6 hydroxyl of PG’s *N-*acetylmuramic acid (MurNAc) [11]. WTA is synthesized on the cytoplasmic face of the cell membrane by modifying a membrane-embedded undecaprenyl-phosphate (C_55_-P) carrier. In *Bacillus subtilis* and *S*. *aureus*, the conserved GlcNAc-ManNAc-GroP linkage unit is first synthesized by the sequential action of the TagO, TagA, and TagB enzymes (originally designated TarOAB in *S*. *aureus*). TagO initiates WTA synthesis by transferring GlcNAc from the UDP-activated sugar to the C_55_-P carrier to produce lipid-α[12]. The TagA glycosyltransferase (GT) then appends ManNAc from a UDP-ManNAc donor, producing a C_55_-PP-GlcNAc-ManNAc disaccharide-lipid product (lipid-β) [13, 14].

Linkage unit synthesis is then completed by TagB, which appends a single GroP to lipid-β using a CDP-glycerol substrate that is produced by the TagD enzyme [13]. In *S*. *aureus*, the linkage unit is then primed by TarF, which attaches at least one additional GroP. The TarIJL enzymes then construct the main chain of the polymer by adding 40–60 ribitol-5-phosphate (RboP) units [15–17]. After being modified with GlcNAc by TarM and TarS, the TarGH ABC-like transporter exports the polymer to the cell surface, where it is further modified with D-alanine to tune its electrostatic properties [18]. The assembled polymer is then covalently attached to the cell wall by an LCP ligase, which catalyzes a phosphotransfer reaction that joins WTA via its linkage unit to PG’s MurNAc [19–21]. *B*. *subtilis* also uses functionally analogous enzymes to produce strain-specific GroP (spp. 168) or RboP (spp. W23) WTA polymers. Recent structural studies have begun to reveal the mechanism through which bacteria produce WTA, including how the polymerization is primed (TarF), and how WTA is modified with GlcNAc (TarM and TarS) and attached to the cell wall (LCP ligases) [22–24]. However, it remains unknown exactly how bacteria produce the highly-conserved linkage unit that connects WTA to the cell wall.

The TagA N-acetylmannosamine transferase catalyzes the first committed step in WTA biosynthesis. It is an attractive target for new therapeutics aimed at treating MRSA infections, as *tagA-* strains are attenuated in virulence and re-sensitized to methicillin, imipenem, and ceftazidime. [25–28]. TagA is also a founding member of the WecB/TagA/CpsF family of GTs (PFAM03808; CAZy GT26), which has over 6,000 members [29, 30]. In addition to WTA, these enzymes synthesize a range of important surface-associated and secreted glycopolymers that function as virulence factors, including capsular polysaccharides of Group B Streptococcus (GBS) and the enterobacterial common antigen present in the outer-membrane of *Escherichia coli* and other Gram-negative bacteria [31–33]. Industrially, this family is important as it includes the GumM GT, an essential enzyme in xanthan gum synthesis in *Xanthomonas campestris* [34]. WecB/TagA/CpsF GTs are distinguished by their ability to elaborate membrane-embedded polyprenol substrates, but the molecular basis of their function remains unknown. Here, we report the crystal structure and biochemical studies of TagA from *Thermoanaerobacter italicus*, a close homolog of *S*. *aureus* TagA. Our results reveal that WecB/TagA/CpsF enzymes adopt a unique GT-E fold and shed considerable light onto their mechanism of catalysis. We propose that membrane association activates TagA by triggering a unique dimer to monomer quaternary structural change that facilitates lipid-α recognition and the formation of a catalytically competent active site. The results of these structural and mechanistic studies represent a major advancement in our understanding of WTA biosynthesis that could facilitate the discovery of new antibiotics that work by disrupting the synthesis of this important bacterial surface polymer.

## Results and discussion

### WecB/TagA/CpsF enzymes are structurally novel glycosyltransferases

To gain insight into the mechanism of catalysis, we determined the 2.0 Å crystal structure of the TagA enzyme from *Thermoanaerobacter italicus* (TagA^ΔC^, residues Met1-Gly195) (**Fig 1B**). TagA^ΔC^ contains the highly conserved amino acid region that defines the WecB/TagA/CpsF family (**S1 Fig**), but lacks 49 C-terminal residues that target the protein to the membrane (*see below*). TagA^ΔC^ is much better suited for structural analyses, since unlike the full-length protein, it does not require high concentrations of salt and glycerol to be solubilized. Selenomethionine (SeMet)-labeled TagA^ΔC^ in its apo-form crystallized in the P2_1_ space group as a dimer, with eight molecules per asymmetric unit. The structure was determined using the multiple anomalous dispersion (MAD) method and is well-defined by continuous electron density. The subunits in the dimer are related by two-fold non-crystallographic symmetry and possess similar atomic structures; their heavy atom coordinates can be superimposed with an RMSD of 0.11 Å. All four dimers in the asymmetric unit also superimpose with similarly low RMSD (average of 0.41 Å). Complete data collection and structural statistics are provided in **Table 1**.

**Table 1:** Crystal data collection and structure refinement statistics.

Data collection	SeMet ^Ti^TagA^ΔC^	SeMet ^Ti^TagA^ΔC^-UDP
PDB Code	5WB4	5WFG
Space group	P2_1_	P2_1_
Cell dimensions		
*a*, *b*, *c* (Å)	77.0, 107.8, 89.4	64.9, 104.2, 90.1
α, β, γ (°)	90.0, 98.3, 90.0	90.0, 108.3, 90.0
Resolution (Å)	88.51–2.00 (2.05–2.00)^b^	85.6–2.9 (3.0–2.9)^b^
Wavelength^a^	0.9791	
*R*_merge_ (%)	15.3 (61.8)	7.4 (64.6)
*I* / σ*I*	7.2 (2.5)	7.2 (1.0)
CC_1/2_	98.9 (84.3)	99.5 (59.4)
Completeness (%)	98.1 (92.8)	88.7 (79.1)
Redundancy	4.8 (4.5)	1.6 (1.6)
Wilson B-factor (Å^2^)	21.7	69.7
**Refinement**		
Resolution (Å)	88.51–2.00	33.03–2.90
No. reflections	96634	24132
*R*_work_ / *R*_free_ (%)^c^	21.9/24.6	20.0/24.7
No. atoms	12043	8456
Protein	11591	8216
Ligand/ion	45	216
Water	407	24
*B*-factors (all atoms)	25.0	81.0
Protein	24.9	80.5
Ligand/ion	29.4	102.9
Water	25.9	43.3
R.m.s. deviations		
Bond lengths (Å)	0.010	0.010
Bond angles (°)	1.04	1.14
Ramachandran favored (%)	89.7	90.4
Ramachandran allowed (%)	9.4	8.9
Ramachandran generally allowed (%)	0.9	0.4
Ramachandran outliers (%)	0.0	0.3

^a^ Selenium peak.

^b^ Values in parentheses are for highest-resolution shell.

^c^ R_free_ calculated using 5% of the data.

TagA adopts a unique α/β tertiary structure that differs markedly from previously described GTs [35, 36]. Each protomer consists of eight β–strands and nine α-helices that form two distinct regions (**Fig 1C**). The N-terminal region of TagA is formed by helices H2 to H4 that pack against a β-hairpin constructed from strands β1 and β2, while its larger C-terminal region is comprised of a six-stranded parallel β-sheet (β–strands β4, β3, β8, β7, β5, and β6) that is surrounded by seven helices (helices H1, H3, and H5 to H9). The arrangement of the six parallel strands forming the β–sheet resembles a Rossmann fold commonly found in nucleotide-binding proteins. The regions are interconnected, with the N-terminal β-hairpin forming a single backbone-backbone hydrogen bond to strand β4 within the C-terminal region (between the amide of Asp13 (β2) and the carbonyl of Asn63 (β3)). In the dimer, the C-terminal helix H8 in one subunit packs against helices H2 and H4 located in the N-terminal region of the adjacent protomer, burying 1,212 Å^2^ of solvent accessible surface area to produce a narrow pore (detailed in **S6 Fig**). Analytical ultracentrifugation (AUC) experiments indicate that TagA^ΔC^ dimerizes in solution with relatively weak affinity, with a monomer-dimer dissociation constant (K_d_) of only 7.4 ± 0.7 μM (**S3A Fig**). The modest dimer affinity may in part be due to the fact that the dimer interface is discontinuous (**Fig 1D**).

To determine the biological relevance of the oligomeric interface observed in the crystal structure we analyzed the SeMet-labeled TagA^ΔC^ structure with the Evolutionary Protein-Protein Classifier (EPPIC) program [37]. EPPIC indicates with 99% certainty that the dimeric interface is biologically relevant. Just as SeMet-TagA^ΔC^ crystallizes as a dimer, native TagA^ΔC^ is dimeric in solution (**S4C and S4D Fig**). Based on the crystal structure of the SeMet-labeled TagA^ΔC^ protein the side chains of Val43 and Ala72 reside at the dimer interface, burying 99% and 84% of their surface area (**S6 Fig**). Consistent with the native and SeMet-labeled proteins adopting similar quaternary structures, introduction of V43E and A72R mutations into TagA^ΔC^ disrupts dimerization (**S4C and S4D Fig**). Combined, these results substantiate the conclusion that the dimeric interface visualized by crystallography is biologically relevant and that it is present in solution in the native form of the protein. As WecB/TagA/CpsF enzymes exhibit related primary sequences, it is expected that they will adopt tertiary structures that are similar to that observed for TagA.

Glycosylation reactions catalyzed by GT enzymes play a central role in biology, creating an enormous array of biologically important oligosaccharides and glycoconjugates. Interestingly, the array of enzymatic machinery used to perform glycosylation is surprisingly simple, and only four distinct GT protein folds have been identified that are capable of glycosyltransferase activity (termed GT-A, GT-B, GT-C, and GT-D enzymes) [32, 36, 38]. TagA^ΔC^ differs markedly from all of these enzymes based on its tertiary structure and how its secondary structural elements are arranged. Notably, TagA lacks the canonical Asp-X-Asp motif found in GT-A enzymes that participate in nucleotide binding and it differs substantially from GT-B and GT-C class enzymes that adopt multi-domain structures [32, 36]. Interestingly, TagA does exhibit limited structural homology with DUF1792 (PDB ID: 4PFX), the founding member of the GT-D family that transfers glucose from UDP-glucose onto protein O-linked hexasaccharides [38]. The backbone coordinates of a subset of residues within the TagA^ΔC^ and DUF1792 structures can be superimposed with an RMSD of 3.7 Å (**S2A Fig**). However, consistent with these enzymes sharing only 15% sequence identity, the arrangement, number, and topology of their secondary structural elements are distinct (**S2B Fig**). Furthermore, the enzymes have different catalytic mechanisms, as TagA exhibits ion-independent glycosyltransferase activity and it lacks the conserved Asp-X-Glu motif present in DUF1792 that coordinates an Mn^2+^ ion cofactor [14]. Thus, TagA and related members of the WecB/TagA/CpsF family adopt a novel glycosyltransferase fold, which we term GT-E.

### Active site architecture

**Fig 2:**
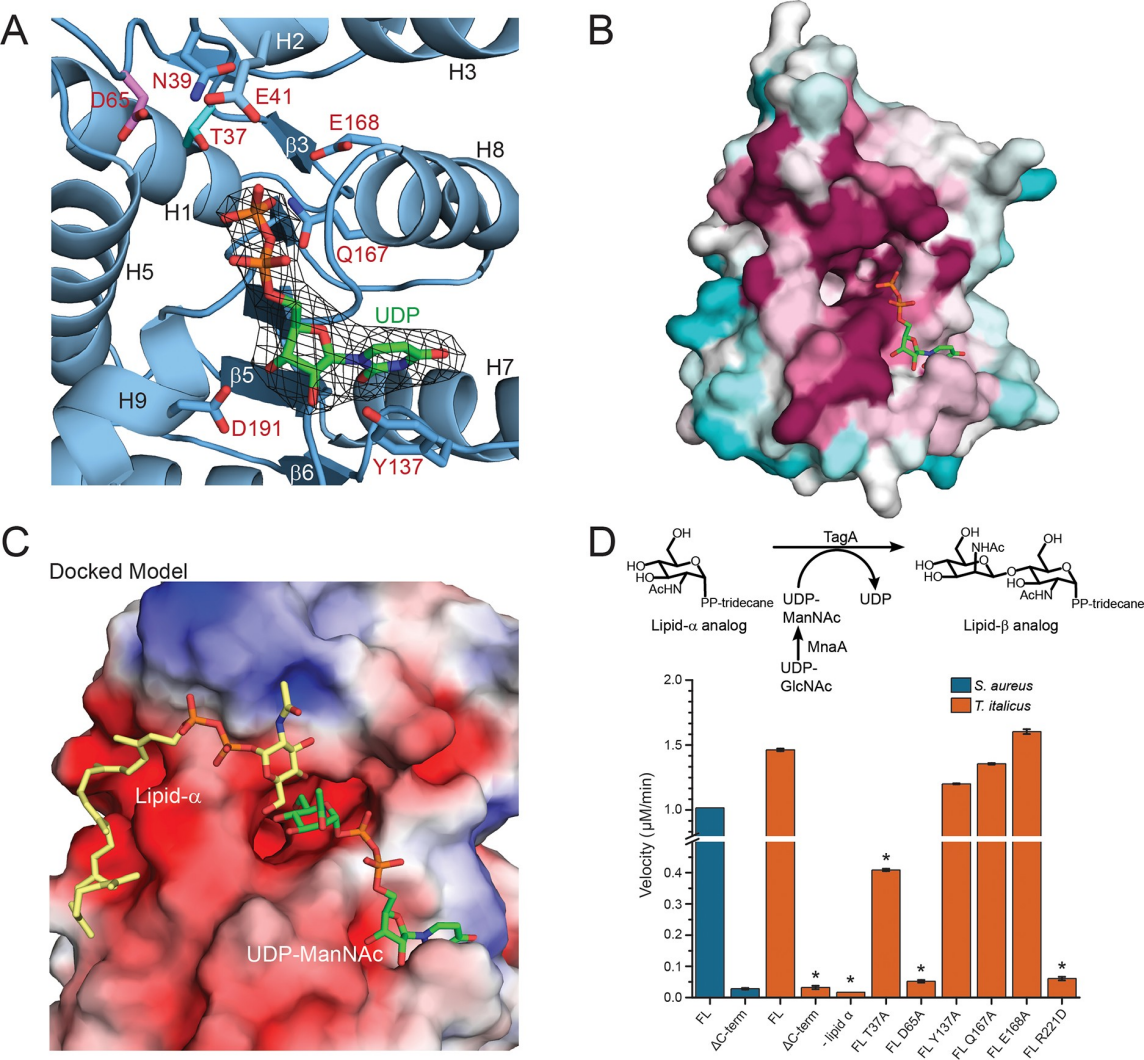
*In silico* substrate binding and mechanistic studies of TagA. (A) Structure of the UDP:TagA^ΔC^ complex. A simulated annealing omit map contoured at 3σ reveals that the β-phosphate of UDP (*orange*) in the UDP:TagA^ΔC^ structure is adjacent to the proposed catalytic base, Asp65 (*pink*), and putative ManNAc stabilizing residue, Thr37 (*cyan*). The uracil nucleoside is pi-stacked over Tyr137. (B) Consurf analysis reveals that UDP projects its phosphates (*orange*) into the pocket and is coordinated by a pocket of highly conserved residues. Highly conserved (*magenta*), moderately conserved (*white*) and weakly conserved (*teal*) residues are indicated. (C) *In silico* generated model of TagA^ΔC^ bound to its substrates. The model contains a lipid-α analog (*yellow*) and UDP-ManNAc (*green*). The electrostatic surface of the protein is shown. Negatively charged residues (*red*), neutral residues (*white*), positively charged residues (*blue*) are shown. The coordinates of the model were generated using a two-step procedure. First, the coordinates of the ligand-bound UDP:TagA^ΔC^ crystal structure were used to restrain the positioning of UDP-ManNAc. Autodock vina was then used to dock lipid-α. The docking results positioned the non-reducing end of GlcNAc toward the C4 in ManNAc when bound to the TagA^ΔC^ dimer. (D) The upper panel indicates the reaction scheme used for the *in vitro* TagA activity assay. 200 nM TagA enzyme was incubated at 30°C with 100 μM lipid-α substrate analog and UDP-ManNAc produced *in situ* from UDP-GlcNAc by the epimerase MnaA, followed by quenching with 4M urea. Conversion of UDP-ManNAc to UDP is monitored at 271 nm using a DNAPak PA200 anion exchange column. The lower panel indicates activity measurements of *T*. *italicus* and *S*. *aureus* TagA enzymes from the *in vitro* TagA activity assay, as described above. Reactions with error bars were performed in triplicate, and asterisks indicate p<0.005 by Student’s T-test.

To define the enzyme active site, we determined the structure of the SeMet-labeled TagA^ΔC^:UDP complex at 3.1 Å resolution (**Fig 2A**). The crystal structure visualizes the enzyme-product complex, as steady-state kinetics studies of *B*. *subtilis* TagA have shown that the enzyme operates via an ordered Bi-Bi mechanism in which UDP-ManNAc binds first and UDP is released last [14]. The complex crystallizes as a dimer of trimers in the P2_1_ space group, with six molecules in each asymmetric unit (**S5 Fig**). The coordinates of the apo- and UDP-bound forms of TagA^ΔC^ are nearly identical (RMSD of 0.18 Å), suggesting that the enzyme binds UDP through a lock-and-key mechanism. In each protomer, UDP contacts the β7-H8 motif within the Rossmann-like fold, as well as the C-terminal edge of strand β5 (**Fig 2A**). The uracil base is engaged in pi stacking with Tyr137 (β6-H7 loop), while Asp191 (H9) contacts the ribose sugar. The trimeric oligomer observed in the structure of the complex is presumably an artifact of crystallization, as AUC experiments using the native TagA^ΔC^ performed in the presence of saturating amounts of UDP indicate that similar to apo-TagA^ΔC^, the UDP-bound protein fits best to a monomer-dimer equilibrium model rather than monomer-trimer or dimer-trimer equilibrium models (K_d_ = 3.5 ± 0.4 μM in the presence of 10:1 UDP:TagA) (**S3A Fig**). Moreover, an EPPIC analysis of the trimer structure indicates that its interfaces are unlikely to be biologically relevant (14% confidence level). In the complex, each UDP molecule is primarily contacted by a single subunit. However, the C2 hydroxyl group in each UDP molecule forms a hydrogen bond with the backbone carbonyl of Val192 (H9) located in the adjacent subunit, which may explain why the complex crystallized as a trimer.

Intriguingly, positioned immediately adjacent to the UDP binding site on each protomer is a large pocket that harbors several phylogenetically conserved amino acids that are important for catalysis (**Fig 2B**). The pocket resides near the C-terminal end of the parallel β3 and β8 strands and has walls that are formed by residues located in helices H5 and H8, as well as residues within the polypeptide segments that connect strand β7 to helix H8, and strand β3 to helix H2. Several highly-conserved residues are located within the pocket and its periphery, including Thr37, Asn39, Asp65, Arg83, Gln167, and Glu168 (**Fig 2A** and **S1 Fig**). Interestingly, in the crystal structure of the TagA^ΔC^:UDP complex, the β-phosphate group of UDP extends inward toward the pocket. As UDP is a competitive inhibitor of the UDP-ManNAc substrate, it is likely that these ligands bind to the same site on the enzyme, such that the ManNAc moiety within UDP-ManNAc is projected into the pocket where it can interact with the conserved side chains of residues Thr37, Gln167 or Glu168 [14]. In an effort to better understand how TagA processes its substrates, we modeled how lipid-α and UDP-ManNAc might bind to TagA. Binding of UDP-ManNAc was modeled using the coordinates of the TagA^ΔC^:UDP crystal structure to position the uracil and ribose components, enabling manual placement of the ManNAc moiety. Lipid-α was then docked *in silico* using Autodock vina (see Materials and Methods*)*. These docking experiments suggest that the UDP-ManNAc and lipid-α substrates bind to opposite sides of the conserved pocket [39]. In models of the enzyme-substrate ternary complex, the GlcNAc and diphosphate portion of lipid-α are positioned near residues that connect strands β4 to helix H4, while the undecaprenyl chain of lipid-α exits near the C-terminus of the TagA^ΔC^ protomer (**Fig 2C**). In this binding mode, the sugar acceptor’s C-4 hydroxyl group is positioned near the side chain of Asp65, while the highly-conserved side chains of Arg83 and Asn39 are adjacent to lipid-α’s diphosphate and GlcNAc, respectively.

To investigate the importance of conserved pocket residues, we reconstituted its GT activity *in vitro* using UDP-ManNAc and a lipid-α analog that replaces its undecaprenyl chain with tridecane, as previously reported [14, 40]. The full-length TagA enzymes from *T*. *italicus* and *S*. *aureus* exhibit similar transferase activities *in vitro*, producing the UDP product at a rate of 1.5 μM min^-1^ and 1.0 μM min^-1^ at 200 nM enzyme concentration, respectively (**Fig 2D**). This is expected, as all TagA homologs presumably catalyze the synthesis of the ManNAc(β1→4)GlcNac glycosidic bond within the conserved linkage unit. Thr37Ala and Asp65Ala mutations in TagA cause the largest decreases in activity (0.41 μM min^-1^ and 0.052 μM min^-1^, respectively), compatible with these residues residing within the enzyme’s active site. As discussed later, the carboxyl side chain of Asp65 is poised to function as general base that deprotonates the nucleophilic C4 hydroxyl group in GlcNAc, while the side chain of Thr37 may stabilize the orientation of ManNAc by interacting with its C5 hydroxyl group.

### A conserved C-terminal appendage is required for catalysis

Surprisingly, the mutational analysis reveals that only the full length TagA protein is enzymatically active *in vitro*, while the truncated TagA^ΔC^ protein used for crystallography and modeling is catalytically inactive (**Fig 2D**). This is compatible with the high level of primary sequence conservation of C-terminal residues within WecB/TagA/CpsF enzymes and suggests that the deleted appendage may be necessary to construct a catalytically competent active site (**S1 Fig**). To gain insight into the function of the appendage, we utilized the structure of the TagA protein modeled using Generative Regularized Models of Proteins (GREMLIN), a recently developed protein modeling server that predicts tertiary structure by exploiting sequence conservation and amino acid co-evolutionary patterns [41, 42]. Only GREMLIN-predicted structures in which TagA was assumed to be monomeric yielded favorable results, and is substantiated by important correlations between residues within the body of TagA^ΔC^ and the appendage (in *T*. *italicus*: Arg83 and Arg205, Ala72 and Val235, Gln47 and Lys234, Val197 and Glu218/Lys217, and Lys198 and Lys217 couplings). In general, the tertiary structures of the GREMLIN-predicted model of monomeric TagA (TagA^GM^) and the experimentally determined crystal structures of TagA^ΔC^ are similar. However, only in TagA^GM^ is the C-terminal appendage present, which forms three α–helices (H10 to H12) that pack against the active site harboring the catalytically important Asp65 and Thr37 residues. The C-terminal appendage is presumably unstructured in dimeric forms of the enzyme, as, importantly, the contact surface used to engage the appendage is occluded by inter-subunit interactions in the crystal structures of TagA^ΔC^.

**Fig 3:**
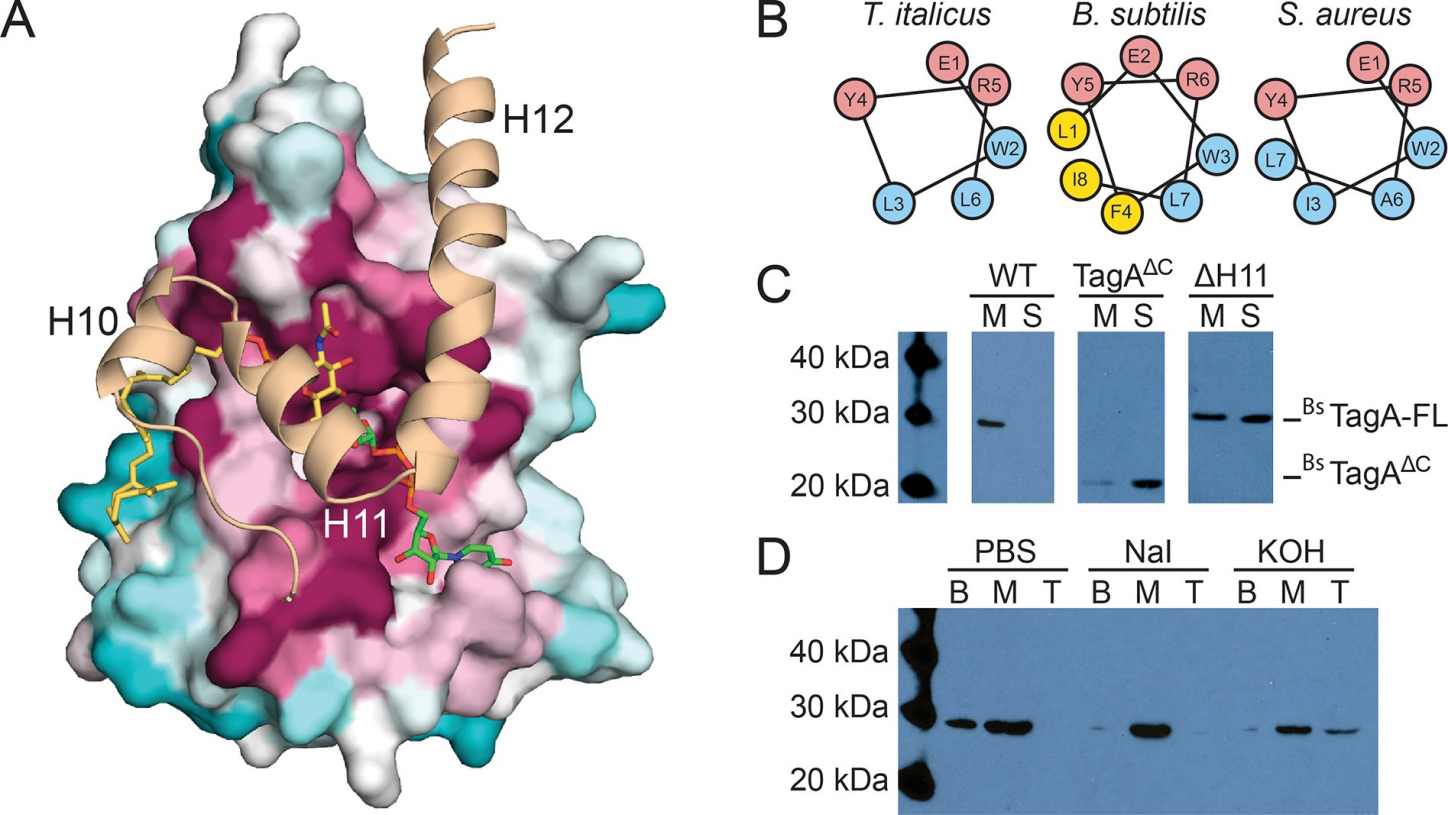
Computational and biochemical studies of the TagA enzyme inform cellular localization. (A) A model of full-length TagA was constructed with experimentally determined TagA^ΔC^ (*surface representation*) and the C-terminal domain, which was modeled by GREMLIN structural prediction (*cartoon representation*). Three C-terminal helices appear to complete the active site and obstruct the dimeric interface of TagA^ΔC^. Highly conserved (*magenta*), moderately conserved (*white*) and weakly conserved (*teal*) residues are indicated for the TagA^ΔC^ crystal structure. (B) Helical wheel projections of helix H11 in TagA homologs predict a putative amphipathic helix. (C) TagA associates with the bacterial cell membrane. Immunoblots of cellular fractionation indicate that *B*. *subtilis* TagA is exclusively localized to the membrane (*M*), while TagA^ΔC^ is primarily localized in the supernatant (*S*). Samples were fractionated by ultracentrifugation identically and the ^Bs^TagA-FL blot was exposed for 10 minutes, the ^Bs^TagA-V196 blot was exposed for 1 minute, and the ^Bs^TagA-ΔH11 blot was exposed for 30 seconds. (D) TagA is a peripheral membrane protein. Chaotropic and alkaline treatments of *B*. *subtilis* TagA reveal that the enzyme is peripherally associated with the membrane and is more effectively displaced by alkaline treatment. Treated membrane fractions were loaded onto a sucrose cushion, centrifuged, and carefully separated into bottom (*B; pellet*), middle (*M; sucrose cushion volume*), and top (*T; sample volume*).

**Fig 4:**
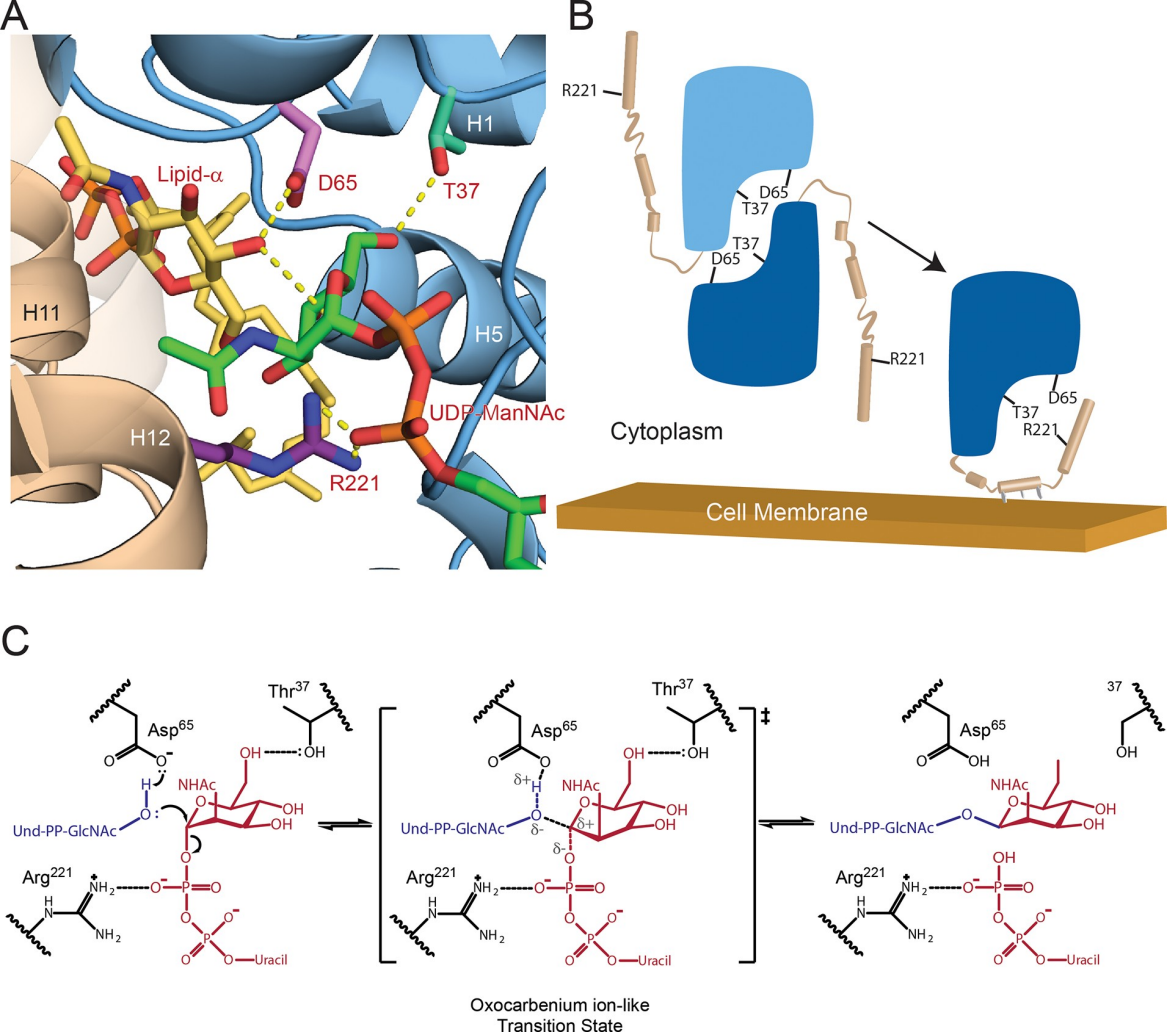
Model of the TagA enzyme-substrate complex and mechanism of catalysis. (A) The proposed active site of TagA co-localizes residues D65, T37, and R221. Lipid-α (*yellow*) is activated by the catalytic base D65 (*pink*), while ManNAc (*green*) is positioned by contacts between its C6 hydroxyl and T37 (*cyan*). The C-terminal helices (*tan*) are modeled according to GREMLIN structural predictions and place R221 (*purple*) adjacent to the phosphates of UDP-ManNAc (*orange*) to putatively stabilize the leaving group. (B) The TagA molecular mechanism is proposed to utilize a dimer to monomer transition to regulate glycosyltransferase activity. TagA is stabilized as a soluble dimer. Upon interaction with the cell membrane, the C-terminus adopts an ordered state and disrupts the dimer interface, which produces a competent active site by co-localizing D65, T37, and R221 to coordinate the soluble UDP-ManNAc and membrane-bound lipid-α substrates. (C) TagA reveals the catalytic mechanism of the GT26 family. Asp65 activates lipid-α, which proceeds to attack UDP-ManNAc in an S_N_2-like mechanism. Coordination between Arg221 and the phosphates of UDP stabilize the leaving group, permitting the oxocarbenium ion-like transition state. The mechanism is completed by glycosidic bond-formation between GlcNAc and ManNAc to form lipid-β.

The model of the TagA^GM^ monomer provides insight into why the C-terminal appendage is critical for catalysis, as it predicts several highly-conserved arginine residues project their side chains into the enzyme’s active site (Arg214, Arg221 and Arg224) (**Fig 3A**). Of particular interest is the side chain of Arg221 within helix H11 of the appendage, as modeling of TagA^GM^ bound to its substrates suggests that the Arg221 guanidino group may stabilize the β-phosphate of the UDP leaving group (**Fig 4A**). Indeed, Arg221Glu mutation in TagA significantly abates TagA GT activity, strongly suggesting that only the monomeric form of TagA is enzymatically active (**Fig 2D**). While not tested in this study, the side chains of Arg214 and Arg224 may also be important for catalysis, as some cation-independent GTs use more than one positively charged residue to stabilize phosphotransfer reaction intermediates [43].

### Membrane-induced structural changes likely facilitate the recognition of bilayer-embedded polyprenol substrates

To build the linkage unit, TagA should associate with the cytoplasmic membrane where it attaches ManNAc to its lipid-α substrate. Intriguingly, only the monomeric form of TagA contains a non-polar surface patch that is suitable for interacting with the lipid bilayer. In monomeric TagA^GM^, helices H11 and H12 within the C-terminal appendage project several non-polar side chains into the solvent for potential membrane interactions (e.g. Leu212, Ile216, and Ile233, in *T*. *italicus* TagA) (**Fig 3B**) [44–48]. To investigate the role of the C-terminal appendage in membrane binding, we determined how this structural element affected TagA localization in *B*. *subtilis*, since unlike *T*. *italicus*, robust tools are available to genetically manipulate this model Gram-positive bacterium. Importantly, the TagA homologs in these organisms are related (34% sequence identity) and both contain the conserved C-terminal appendage (**S1 Fig**). *B*. *subtilis* cells expressing hexahistidine-tagged TagA (^Bs^TagA) were fractionated and analyzed by Western blotting. ^Bs^TagA peripherally associates with the membrane, since it is present in the membrane fraction, but released into the soluble fraction after adding either potassium hydroxide or the chaotropic salt sodium iodide (**Fig 3C** and **3D**, respectively). Interestingly, TagA lacking the C-terminal appendage (^Bs^TagA^ΔC^, residues Met1-Val196) primarily partitions into the soluble fraction, indicating that this structural element tethers the protein to the membrane (**Fig 3C**).

To identify specific residues required for peripheral membrane binding and to validate the structural model of the full-length TagA^GM^ protein, we constructed a ^Bs^TagA mutant that replaces surface-exposed hydrophobic residues with hydrophilic substitutes within helix H11 (^Bs^TagA^ΔH11^, ^Bs^TagA containing L208Q, F211K, L215E mutations, yellow colored residues in **Fig 3B**). Fractionation studies reveal that ^Bs^TagA^ΔH11^ is significantly more solubilized into the cytoplasm compared to wild-type ^Bs^TagA, which is exclusively found in the membrane (**Fig 3C**). These results substantiate the predicted structure of the monomer and suggest that monomeric TagA associates with the membrane via surface-exposed non-polar residues located within helix H11. Other bacterial enzymes are targeted to the membrane via terminal helices (e.g. TagB, FtsA, MinD, PBP enzymes), but TagA is novel because its helices likely form an integral part of the active site upon membrane association [44–48].

Biochemical studies suggest that prior to engaging the membrane, full-length TagA in the cytoplasm is primarily dimeric and in equilibrium with its monomeric form. This is supported by chemical crosslinking studies of cells expressing *T*. *italicus* TagA that show the enzyme exists as a mixture of dimeric and monomeric species (**S3C Fig**). It is also substantiated by SEC experiments that reveal the full-length protein is primarily dimeric in aqueous solvent, but that monomeric and higher-order species are also present (**S4A and S4B Fig**). Interestingly, our results reveal that this equilibrium can be shifted toward the monomeric form by reducing the hydrophobicity of the membrane-targeting C-terminal appendage. We constructed a full-length *T*. *italicus* TagA mutant that contained four amino acid substitutions: I203E, L209Q, L212K and I216E. Based on the TagA^GM^ model, these alterations are expected to increase the polarity of the membrane-targeting patch that is formed by helices H10-H12. Unlike the dimeric native protein, the mutant is primarily monomeric according to SEC (**S4A and S4B Fig**). This finding is compatible with native TagA in aqueous solution being in equilibrium between dimeric and monomeric forms. The mutations shift the equilibrium toward the monomeric state, presumably by reducing the entropic cost associated with clustering these non-polar residues together, which is only expected to occur in the monomer. Thus, we conclude that in the cell, TagA toggles between at least two distinct states: 1) membrane associated enzymatically active monomers, in which the C-terminal appendage contributes Arg221 to the active site to facilitate catalysis, and 2) inactive dimers in the cytoplasm, in which inter-subunit interactions mask an incompletely formed active site.

Collectively, our data suggest that TagA is activated through a unique membrane-association mechanism that is mediated by residues at its C-terminus. TagA is in equilibrium between monomeric and dimeric states. Removed from the membrane, it is primarily dimeric with interfacial interactions obstructing the binding site for its C-terminal appendage and holding the enzyme in an inactive, dimeric state (**Fig 4B**). However, upon encountering the membrane, we posit that non-polar side chains within the appendage are inserted into the bilayer, thereby nucleating its folding and the formation of a monomeric, catalytically functional enzyme in which the appendage contributes key active site residues. In the TagA^GM^ monomer, a gap is located between helices H11 and H12 in the C-terminal appendage, forming a short pore that connects the active site to the protein’s membrane binding surface. Thus, membrane induced folding of the C-terminal appendage may also facilitate recognition of the lipid-α by providing an additional binding site for several of its non-polar prenyl groups. While the catalytic activities of other membrane-associated enzymes are regulated by lipid bilayer-induced quaternary structural changes or allosteric mechanisms, to the best of our knowledge, the TagA activation mechanism outlined here is unique [49].

In conclusion, our results provide direct insight into how TagA enzymes synthesize the conserved linkage unit used to attach WTA to the cell wall and, more generally, how members of the large WecB/TagA/CpsF GT family produce a range of important surface-associated and secreted bacterial glycopolymers. TagA is a structurally unique GT that we propose defines a new GT-E fold. Removed from the membrane, it forms catalytically inactive dimers that are presumably incapable of mediating spurious GT reactions or hydrolyzing UDP-ManNAc. However, upon encountering the membrane containing its lipid-α substrate, conserved C-terminal residues in TagA fold into an essential active site appendage, stabilizing the monomeric and catalytically-active form of the enzyme. From our structural and biochemical data, a working model of the catalytic mechanism can be proposed (**Fig 4C**). Previous studies have shown that catalysis likely occurs via an S_N_2-like mechanism that inverts the anomeric stereochemistry of ManNAc [14]. It seems likely that Asp65 functions as a base, deprotonating the GlcNAc C4 hydroxyl in lipid-α. This would facilitate its nucleophilic attack at the anomeric carbon of ManNAc, resulting in an oxocarbenium ion-like transition state that is stabilized by electrostatic interactions between Arg221, donated by the C-terminal appendage, and the diphosphate moiety of UDP. Thr37 within the conserved pocket may play an important role in orienting the ManNAc moiety of the sugar donor, poising its electrophilic center for glycosidic bond formation. A complete understanding of the catalytic mechanism will require the structure determination of additional key reaction intermediates. As WTA and other bacterial glycopolymers are critical components of the cell wall, the results of these studies are of fundamental importance and could facilitate the discovery of GT-E (WecB/TagA/CpsF) specific enzyme inhibitors that could be useful antibiotics.

## Materials and methods

### Cloning, expression, protein purification, and crystallization

Bacterial strains and plasmids used in this study are listed in SI Materials and Methods, Table I. Protocols for protein purification, crystallization, and structure determination are detailed in SI Materials and Methods.

### Mutagenesis and activity assays

Conservation analysis was conducted using the Consurf Server [50–52]. The lipid-α or UDP-ManNAc substrates were generated *in silico* using the Phenix electronic Ligand Builder and Optimization Workbench (Phenix.eLBOW) [53]. Cis-trans configuration and stereochemistry were confirmed or corrected using the Phenix Restraints Editor Especially Ligands (Phenix.REEL). The substrates were docked to the 2.0 Å resolution protomer structure using Autodock vina with a 25 x 25 x 25 Å search space and an exhaustiveness of 18 [39]. TagA *in vitro* enzyme activity was determined using an anion-exchange HPLC system and the following assay conditions: 0.2 μM TagA; 100 μM lipid-α; 100 μM UDP-GlcNAc; 3 μM MnaA; 50 mM Tris-HCl, pH 7.5; and 250 mM NaCl [40]. Reactions were incubated for 40 minutes before quenching with 3 M urea. The reactions were separated with a DNAPak PA200 anion-exchange column using a buffer gradient of 100% Buffer A (20 mM NH4HCOO3, and 10% MeCN, pH 8.0) to 90% Buffer A plus 10% Buffer B (20 mM NH4HCOO3, 10% MeCN, and 1 M NaCl, pH 8.0) over 10 minutes. UDP-ManNAc or UDP elution peaks were monitored at 271 nm and integrated to determine turnover rate.

### Cell fractionation

Overnight *B*. *subtilis* cultures containing selective antibiotics were diluted 1:100 into 1 L of fresh LB broth containing 1 mM IPTG. Cultures were incubated at 37°C and 250 rpm until an OD_600_ of 1.0 was reached. Cells were pelleted at room temperature, washed once with PBS, and then frozen at -80°C. Cells were fractioned as previously reported with several exceptions [48]. Cells were re-suspended in 10 mL of Lysis Buffer (PBS, pH 7.3; 1 mM EDTA; 1 mM DTT; 10 μg/mL RNase; 10 μg/mL DNase; 2 mM PMSF, and 100 μL Protease inhibitor cocktail) and sonicated on ice for eight minutes, with one minute “on” (one second pulses for 60 seconds) and one minute “off” (no pulses for 60 seconds). Ten milliliters of lysate were divided into two 5 mL volumes and centrifuged in a Beckman type 50 TI rotor in a Beckman XPN-100 preparative ultracentrifuge. The lysate was centrifuged at 9,600 rpm for 10 minutes to remove cellular debris. The supernatant was collected and centrifuged at 21,000 rpm. The supernatant was removed and spun for a third time at 40,000 rpm for one hour. This final supernatant fraction was considered to be the soluble fraction, and the remaining pellet, which contained the membrane fraction was re-suspended in 1 mL of ice-cold Lysis Buffer. The membrane fraction was diluted ten-fold for comparison with the supernatant and samples were run on an SDS-PAGE gel for 50 minutes at 170 V and transferred to a PVDF membrane using an iBlot transfer device (ThermoFisher Scientific). The membrane was fixed in methanol for five minutes, briefly washed in water, and then blocked over-day in TBST Blocking Buffer (20 mM Tris-HCl, pH 7.5; 500 mM NaCl; 0.05% Tween; and 5% w/v nonfat milk). The membrane was washed, incubated with primary antibody (Invitrogen #MA-21215, mouse anti-6_X_His, 1:1000 dilution in TBST Blocking Buffer) overnight at 4°C, washed again, and incubated with secondary antibody (Sigma-Aldrich #A9044, anti-mouse horseradish peroxidase). The membrane was incubated with SuperSignal West Pico Chemiluminescent Substrate (Thermo Scientific #34080) for five minutes and exposed to radiography film for thirty seconds to ten minutes.

### Chaotropic agent analysis

Membrane fractions were prepared as described above and re-suspended to 600 μL. Fractionation was performed as previously reported, with some minor changes [54]. 200 μL of membrane resuspension was diluted 1:3 in PBS containing 1.5 M sodium iodide, 0.1 N potassium hydroxide, or PBS alone. Samples were loaded onto a 2.4 mL sucrose cushion (PBS and chaotrope, 0.5 M sucrose) and centrifuged for 30 minutes at 40,000 rpm. The top (800 μL) and middle (2.4 mL) fractions were removed, and the pellet was re-suspended in 200 μL of PBS. The top and middle fractions were precipitated with 10% trichloroacetic acid on ice for 30 minutes, centrifuged at 20,000 *g* for 10 minutes, and then re-suspended in 200 uL of 8 M urea. Samples were mixed 1:1 with 2X SDS loading dye (100 mM Tris base, 200 mM DTT, 4% SDS, 0.2% bromophenol blue, 20% glycerol). Immunoblotting was performed as described above.

## Supporting information

S1 Fig TagA primary sequence homology. The National Center for Biotechnology Institute’s Basic Local Alignment Tool (BLAST) was used to determine TagA sequence homologs with high sequence identity. The Clustal Omega multiple sequence alignment tool was used to generate a sequence alignment [9]. Secondary structure is shown above the sequence and coloring is indicated in the key.
(TIF)

S2 Fig TagA structural comparison with DUF1792, a GT-D enzyme. (A) TagA aligns with DUF1792 with a DALI Z-score of 7.2 and an RMSD of 3.7 Å. Direct comparison reveals that a β-sheet composed of parallel β-strands is the main component of structural similarity. The tertiary organization of secondary structural elements between TarA and DUF1792 is significantly different. (B) Cartoon representation of secondary structure topology highlights that TagA has fewer β-strands in its sheet than DUF1792 and reinforces the dissimilarity in the order of secondary structural elements.
(TIF)

S3 Fig TagA oligomerization. The dissociation constant for TagA oligomerization was determined by equilibrium sedimentation analytical ultracentrifugation. The concentration distribution of TagA for three rotor speeds (12k, 14k, and 20k rpm) at three protein concentrations (0.99, 0.64, and 0.21 mM) for (A) apo-state TagA and (B) UDP-bound TagA. The lower panel shows the regression residuals for each protein concentration and centrifugal speed. The data were collected at 280 nm at 4°C and referenced against 50 mM Tris-HCl, pH 7.5, and 200 mM NaCl. (C) Crosslinking studies with disuccinimidyl suberate (DSS) in *E*. *coli* cells expressing *T*. *italicus* TagA constructs confirm that a dimer species is formed in the context of the cell. Both TagA and TagA^ΔC^ are monomeric under denaturing conditions (+ DMSO,—DSS); however, the addition of 1 mM DSS (+ DMSO, + DSS) produced a band corresponding to dimer species.
(TIF)

S4 Fig Size exclusion chromatography of full-length and C-terminal truncated TagA. (A) SEC chromatograms of *T*. *italicus* TagA^FL^ (FL WT; full length wild-type, *red*) and TagA^FL^ containing mutations in its C-terminal membrane-targeting appendage (I203E/L209Q/L212K/I216E mutant, *blue*). Based on the monomeric TagA^GM^ model, the four mutations increase the polarity of the hydrophobic surface formed by helices H10 and H11. These non-polar residues form a continuous patch in TagA^GM^ that we have shown to be important for membrane binding. The mutations stabilize formation of the monomer, presumably by reducing unfavorable entropic changes associated with solvating the hydrophobic surface of the native protein. (B) SEC calibration curve used to assign the oligomeric states of WT and mutant forms of TagA. A plot of the log of the molecular weight versus elution volume is shown. Elution position of molecular weight standards are shown in grey and were obtained in a separate experiment. (C) SEC chromatograms of TagA^ΔC^ and mutant forms of TagA^ΔC^: V43E (*orange*) and A72R (*green*). Based on the crystal structure of apo- TagA^ΔC^, these mutations are at the interface and impede dimerization, which we confirm here via SEC. (D) Identical to (B) repeated for consistency.
(TIF)

S5 Fig TagA^ΔC^:UDP complex crystallizes as a dimer of trimers. (A) Cartoon representation of the TagA^ΔC^:UDP complex. (B) Surface representation of the TagA^ΔC^:UDP complex. (C) View of one trimer unit within the crystallographic dimer. The C-termini are projected inward toward the center of symmetry. Green arrows indicate UDP, which can be seen at the interface between protomers of the trimer.
(TIF)

S6 Fig Buried residues at the TagA^ΔC^ dimeric interface. An EPPIC analysis of PDB 5WB4 identified fourteen residues with a buried surface area ≥ 75% in either protomer. An additional three residues engaged in polar bonds with lower percent buried surface area are shown. The side chains of these residues are shown and color-coded as follows: hydrophobic (*yellow*, Ala40, Val43, SeMet44, Phe71, Ala72, Phe87, Ala109, Ala161 and Val192), glycines (*green*, Gly65, Gly163 and Gly187), hydrogen bonds (*orange*, Ser67 and Asp88), polar (magenta, Asp191), and cation-pi stacking interactions (*red*, Lys48 and Tyr137). Mutation of residues Val43 and Ala72 were shown to disrupt dimerization in **S4C Fig**.
(TIF)

S1 Table Strains and plasmids used in this study. (DOCX)

S1 Methods Supplemental methods and references. (DOCX)
