# Supplementary figures and images for: Structure and mechanism of TagA, a novel membrane-associated glycosyltransferase that produces wall teichoic acids in pathogenic bacteria
Source: PLoS Pathog. 2019 Apr 19;15(4):e1007723. doi: 10.1371/journal.ppat.1007723 (PMC6493773; doi:10.1371/journal.ppat.1007723)

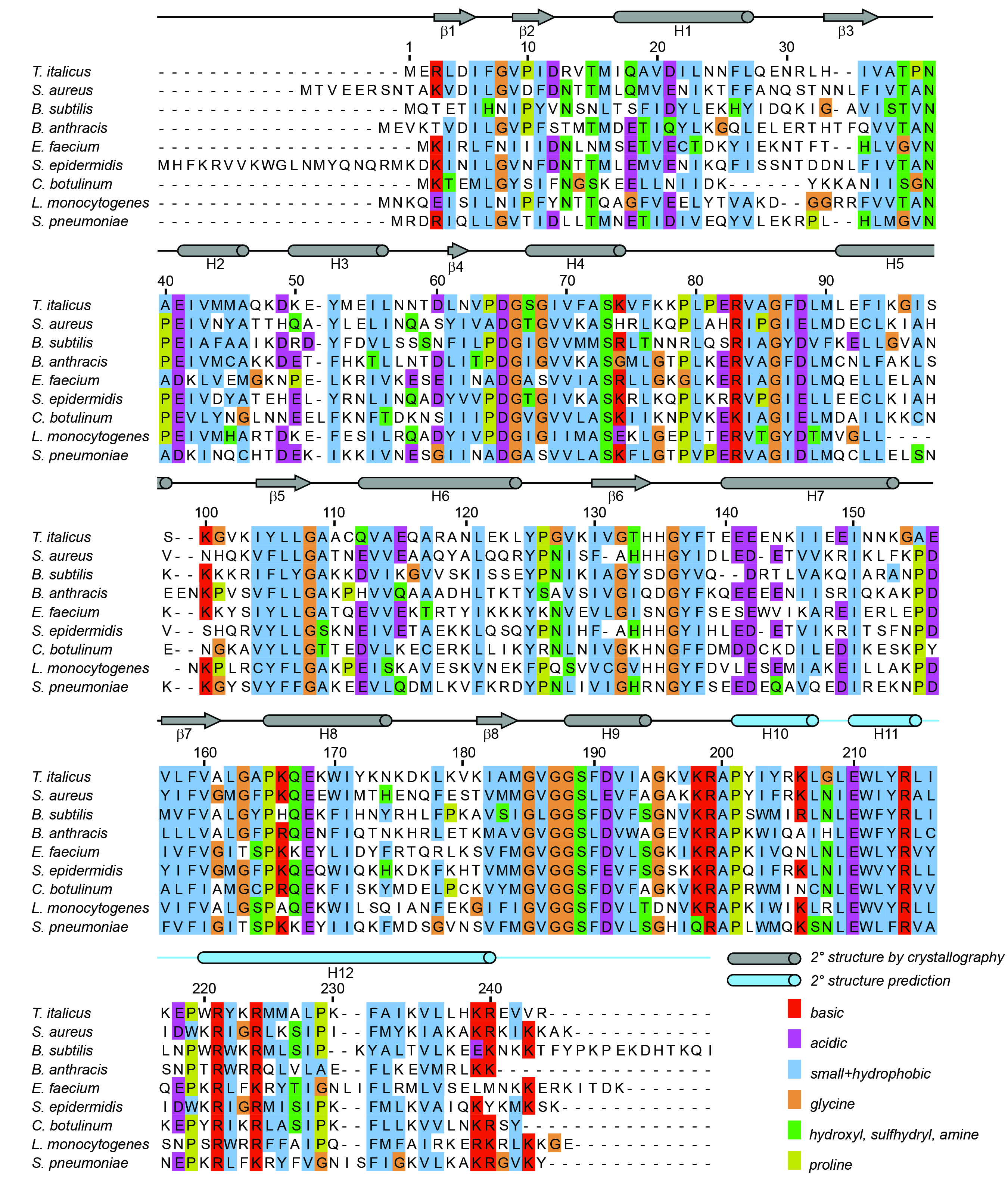

Supplement: S1 Fig — The National Center for Biotechnology Institute’s Basic Local Alignment Tool (BLAST) was used to determine TagA sequence homologs with high sequence identity. The Clustal Omega multiple sequence alignment tool was used to generate a sequence alignment [9]. Secondary structure is shown above the sequence and coloring is indicated in the key. (TIF) [file ppat.1007723.s001.tif]

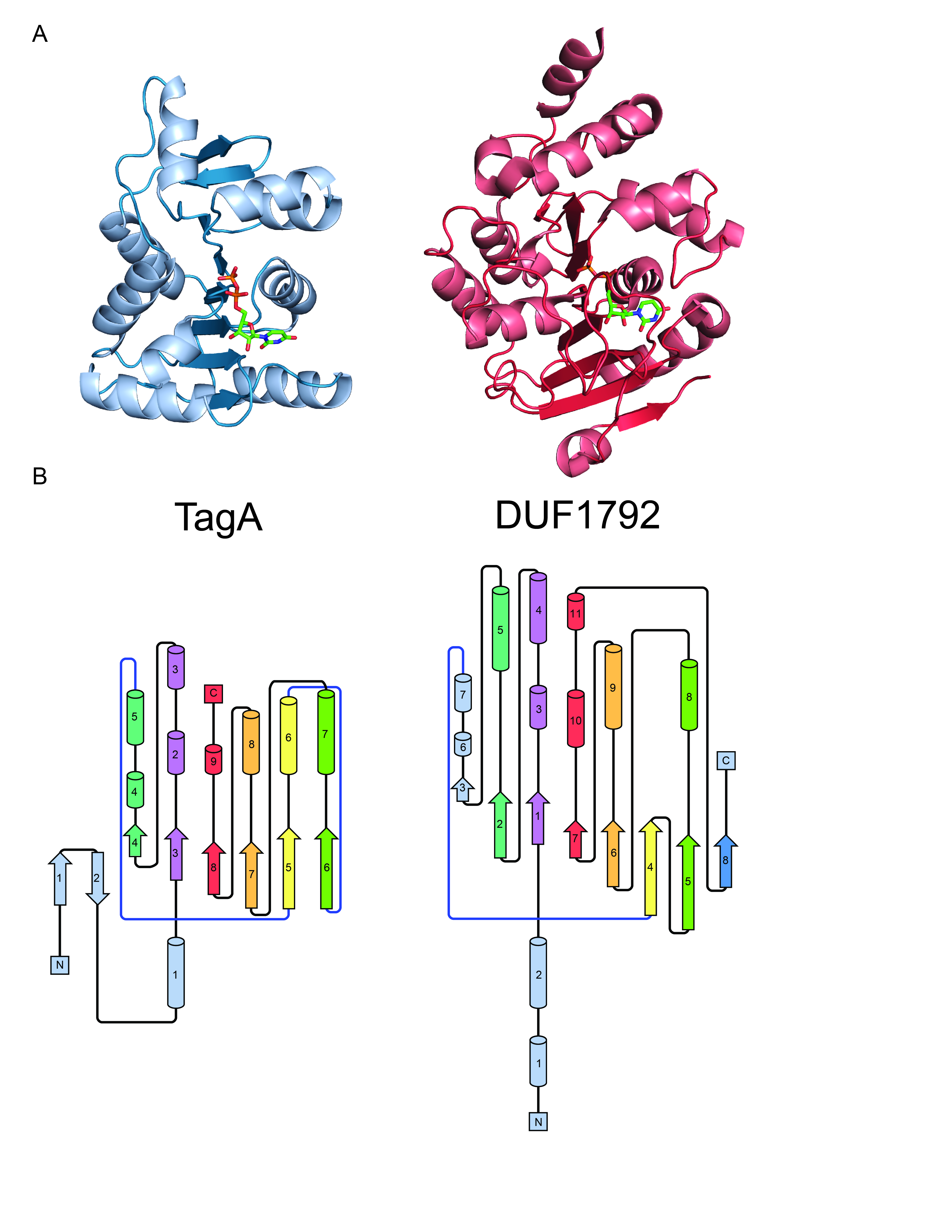

Supplement: S2 Fig — (A) TagA aligns with DUF1792 with a DALI Z-score of 7.2 and an RMSD of 3.7 Å. Direct comparison reveals that a β-sheet composed of parallel β-strands is the main component of structural similarity. The tertiary organization of secondary structural elements between TarA and DUF1792 is significantly different. (B) Cartoon representation of secondary structure topology highlights that TagA has fewer β-strands in its sheet than DUF1792 and reinforces the dissimilarity in the order of secondary structural elements. (TIF) [file ppat.1007723.s002.tif]

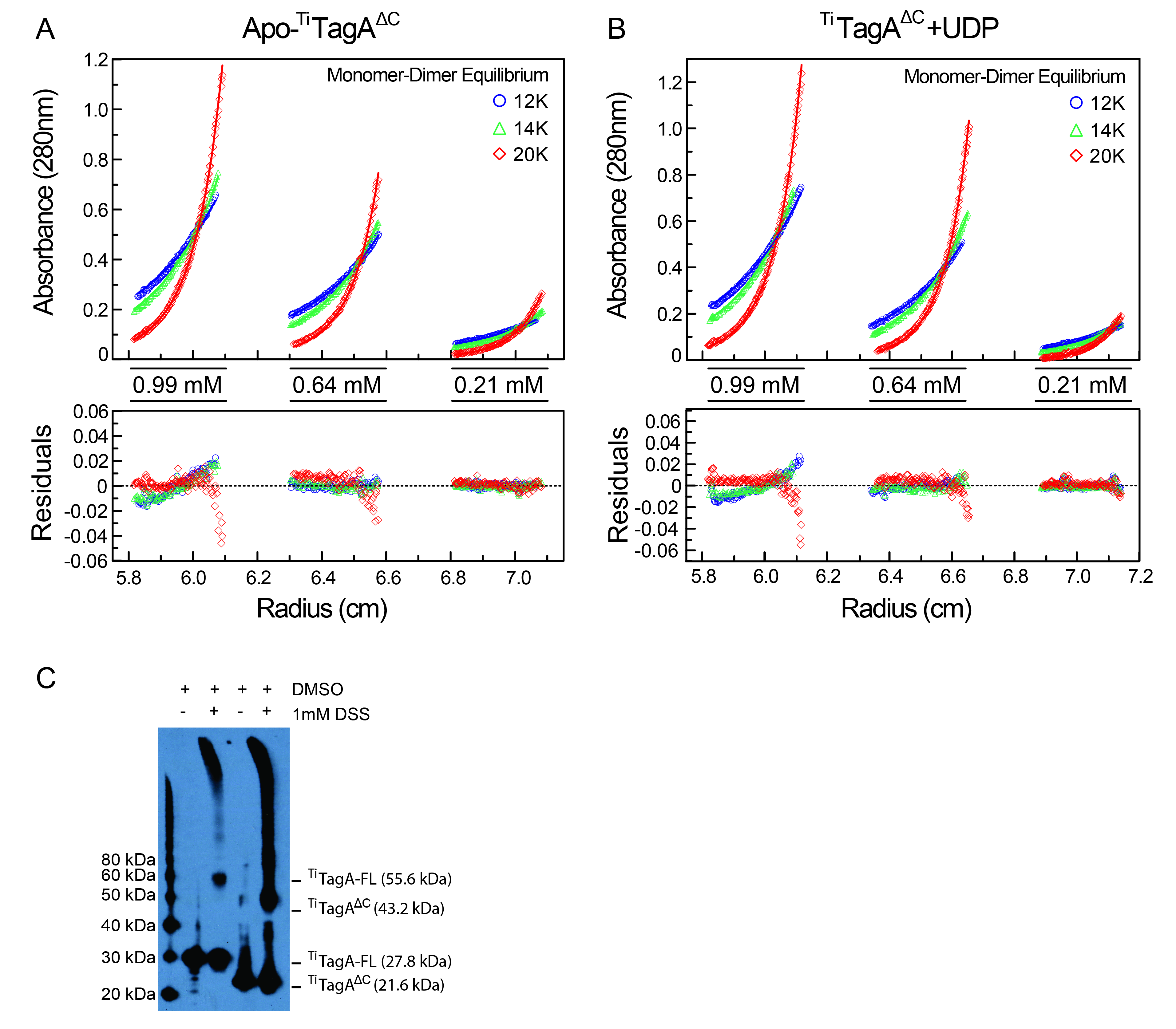

Supplement: S3 Fig — The dissociation constant for TagA oligomerization was determined by equilibrium sedimentation analytical ultracentrifugation. The concentration distribution of TagA for three rotor speeds (12k, 14k, and 20k rpm) at three protein concentrations (0.99, 0.64, and 0.21 mM) for (A) apo-state TagA and (B) UDP-bound TagA. The lower panel shows the regression residuals for each protein concentration and centrifugal speed. The data were collected at 280 nm at 4°C and referenced against 50 mM Tris-HCl, pH 7.5, and 200 mM NaCl. (C) Crosslinking studies with disuccinimidyl suberate (DSS) in E. coli cells expressing T. italicus TagA constructs confirm that a dimer species is formed in the context of the cell. Both TagA and TagAΔC are monomeric under denaturing conditions (+ DMSO,—DSS); however, the addition of 1 mM DSS (+ DMSO, + DSS) produced a band corresponding to dimer species. (TIF) [file ppat.1007723.s003.tif]

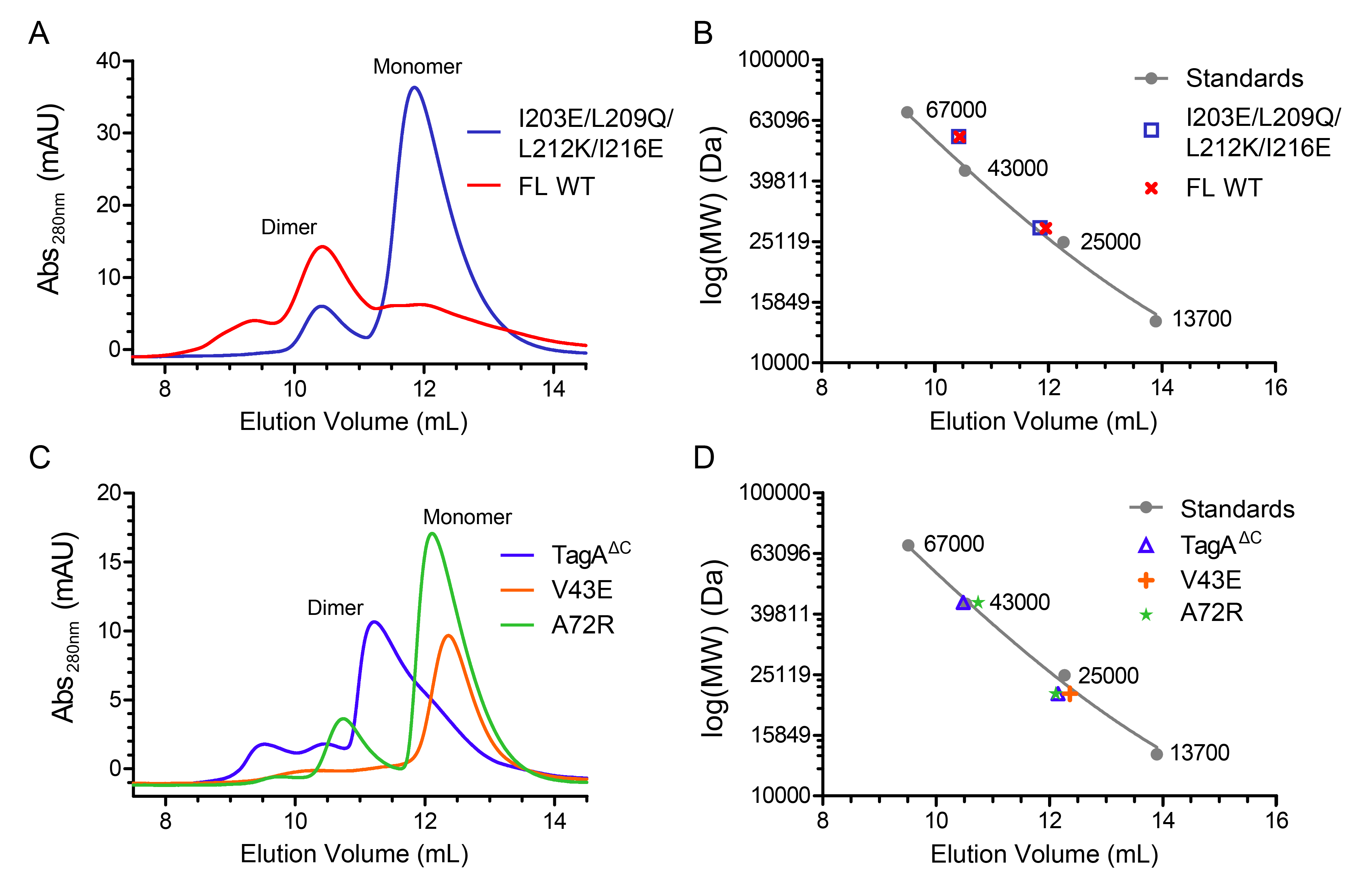

Supplement: S4 Fig — (A) SEC chromatograms of T. italicus TagAFL (FL WT; full length wild-type, red) and TagAFL containing mutations in its C-terminal membrane-targeting appendage (I203E/L209Q/L212K/I216E mutant, blue). Based on the monomeric TagAGM model, the four mutations increase the polarity of the hydrophobic surface formed by helices H10 and H11. These non-polar residues form a continuous patch in TagAGM that we have shown to be important for membrane binding. The mutations stabilize formation of the monomer, presumably by reducing unfavorable entropic changes associated with solvating the hydrophobic surface of the native protein. (B) SEC calibration curve used to assign the oligomeric states of WT and mutant forms of TagA. A plot of the log of the molecular weight versus elution volume is shown. Elution position of molecular weight standards are shown in grey and were obtained in a separate experiment. (C) SEC chromatograms of TagAΔC and mutant forms of TagAΔC: V43E (orange) and A72R (green). Based on the crystal structure of apo- TagAΔC, these mutations are at the interface and impede dimerization, which we confirm here via SEC. (D) Identical to (B) repeated for consistency. (TIF) [file ppat.1007723.s004.tif]

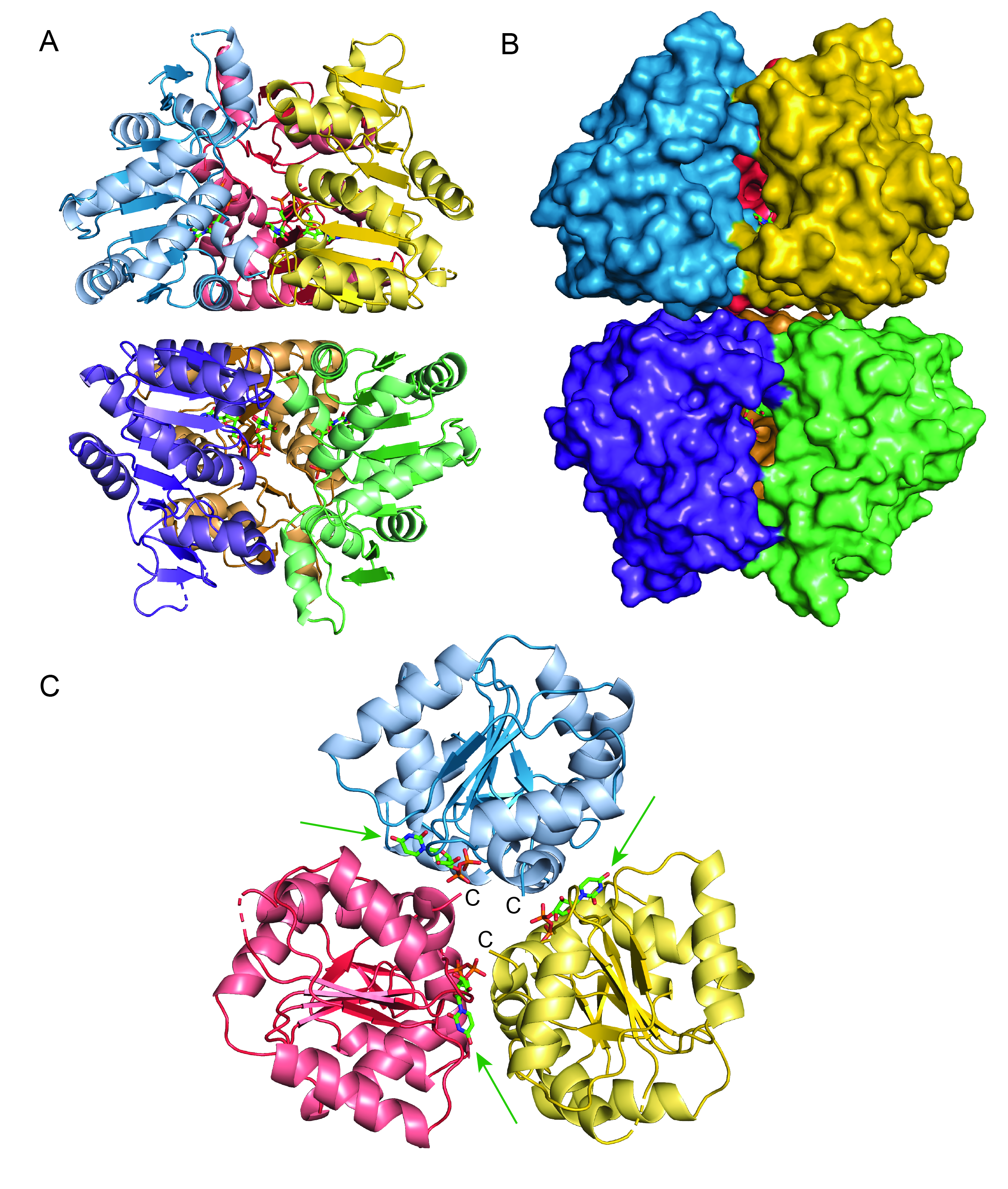

Supplement: S5 Fig — (A) Cartoon representation of the TagAΔC:UDP complex. (B) Surface representation of the TagAΔC:UDP complex. (C) View of one trimer unit within the crystallographic dimer. The C-termini are projected inward toward the center of symmetry. Green arrows indicate UDP, which can be seen at the interface between protomers of the trimer. (TIF) [file ppat.1007723.s005.tif]

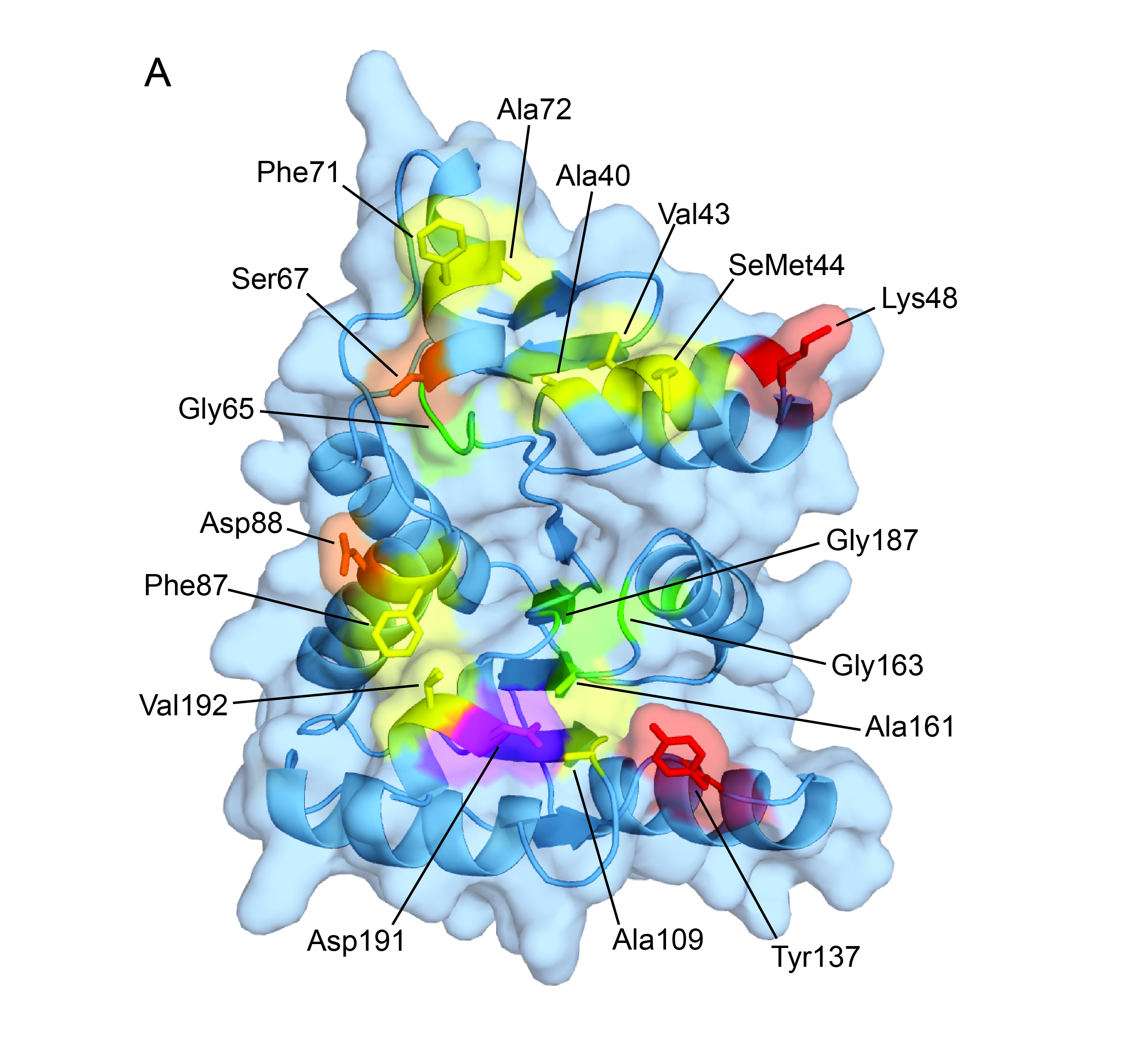

Supplement: S6 Fig — An EPPIC analysis of PDB 5WB4 identified fourteen residues with a buried surface area ≥ 75% in either protomer. An additional three residues engaged in polar bonds with lower percent buried surface area are shown. The side chains of these residues are shown and color-coded as follows: hydrophobic (yellow, Ala40, Val43, SeMet44, Phe71, Ala72, Phe87, Ala109, Ala161 and Val192), glycines (green, Gly65, Gly163 and Gly187), hydrogen bonds (orange, Ser67 and Asp88), polar (magenta, Asp191), and cation-pi stacking interactions (red, Lys48 and Tyr137). Mutation of residues Val43 and Ala72 were shown to disrupt dimerization in S4C Fig. (TIF) [file ppat.1007723.s006.tif]
