# Supplementary material for: Structure and mechanism of TagA, a novel membrane-associated glycosyltransferase that produces wall teichoic acids in pathogenic bacteria
Source: PLoS Pathog. 2019 Apr 19;15(4):e1007723. doi: 10.1371/journal.ppat.1007723 (PMC6493773; doi:10.1371/journal.ppat.1007723)
Supplement: S1 Table — (DOCX) [file ppat.1007723.s007.docx]

**S1 Table: Strains and plasmids used in this study**

| **Strain** | **Genotype/Description** | **Source** |
| --- | --- | --- |
| RC168 | *B. subtilis* Wild type *trpC2* | BGSC |
| RC168tagAFL | RC168, *thrC*::pBL113-^His^*tagA* | Current study |
| RC168tagAFLΔtagA | RC168tagAFL, *thrC*::pBL113-^His^*tagA tagA*::*spec* |  |
| RC168tagAV196 | RC168 *thrC*::pBL113-^His^*TagA-V196* | Current study |
| RC168tagAΔH11 | RC168 *thrC*::pBL113-^His^*TagA*ΔH11 | Current study |
| RC21tagAFL | *E. coli BL21(DE3)* cells with pMAPLe4 containing *T. italicus* TagA | Current study |
| RC21tagAG195 | *E. coli BL21(DE3)* cells with pMAPLe4 containing *T. italicus* TagA (M1-G195) | Current study |
| RC21tagAΔSrf4 | *E. coli BL21(DE3)* cells with pMAPLe4 containing *T. italicus* TagA and I203E, L209Q, L212K, I216E mutations | Current study |
|  |  |  |
| **Plasmid** | **Genotype/Description** | **Source** |
| pMAPLe4 | *E. coli* plasmid that expresses proteins with N-terminal maltose binding protein fusion | [10] |
| pBL113 | *B. subtilis* – *E. coli* shuttle vector derived from pRDC19 (3) that integrates into the thrC locus in the *B. subtilis* genome, Amp^R^, Erm^R^, IPTG inducible | [11] |
| pHisTagAFL | *pBL113-containing B. subtilis tagA* gene with a 5’ hexa-his tag. | Current study |
| pHisTagAV196 | *pBL113-containing B. subtilis tagA* gene with a 5’ hexa-his tag truncated at G618 to produce a V196 translated product | Current study |
| RC21tagAΔSrf4 | *E. coli BL21(DE3)* cells with pMAPLe4 containing *T. italicus* TagA and I203E, L209Q, L212K, I216E mutations | Current study |
| pIC156 | *E. coli/B. subtilis* shuttle vector with spectinomycin^R^ cassette. | [12] |
| p*tagA*::spec | pIC156 with genomic 1kb DNA flanking *tagA* from *B. subtilis* 168 inserted 5’ and 3‘ to spec^R^ cassette | Current study |
