# Supplementary material for: Structure and mechanism of TagA, a novel membrane-associated glycosyltransferase that produces wall teichoic acids in pathogenic bacteria
Source: PLoS Pathog. 2019 Apr 19;15(4):e1007723. doi: 10.1371/journal.ppat.1007723 (PMC6493773; doi:10.1371/journal.ppat.1007723)
Supplement: S1 Methods — (DOCX) [file ppat.1007723.s008.docx]

**Supporting Information Methods**

Cloning, expression, and protein purification. The N-terminal domain of TagA from *T. italicus* (^Ti^TagA^ΔC^, residues Met1-Gly195) or *S. aureus* (^Sa^TagA^ΔC^, residues Ala10-Ala204) was expressed from a pMAPLe4 plasmid in *Escherichia coli* BL21(DE3) cells (Table S1). Standard methods were employed, with cultures grown in the presence of 50 µM kanamycin at 37°C until an OD_600_ of 0.6-0.8 was reached. Protein expression was initiated by adding isopropyl-β-D-1-thiogalactopyranoside (IPTG) to 1 mM, followed by overnight protein expression at 18°C. A four-liter cell culture was harvested by centrifugation at 7000 rpm in a Beckman JA-10 rotor, and the pellet was re-suspended in 40 mL of Buffer A (50 mM Tris, pH 7.5; 500 mM NaCl; 40 mM CHAPS) with 400 µL of protease inhibitor cocktail (Sigma) and 2 mM phenylmethanesulfonylfluoride (PMSF). The cells were then lysed using an EmulsiFlex high pressure homogenizer (Avestin). Cell lysates were fractionated by centrifugation at 15,000 rpm in a Beckman JA-20 rotor, and the soluble portion was applied to a gravity column containing 10 mL of suspended His-Pure Co^2+^ resin (Life Technologies) that was pre-equilibrated with Buffer A. The resin was washed with 20 mL aliquots of Buffer B (50 mM Tris, pH 7.5; 500 mM NaCl; 0.5% CHAPS) that contained 0, 25, or 50 mM imidazole. His-tagged TagA^ΔC^ was eluted using Buffer B with 500 mM imidazole, and the fractions were pooled and concentrated using a 10 kDa MWCO Amicon Ultra-15 centrifugal filter (Millipore). To remove His_6_-tag from the protein, TEV protease was added to TagA^ΔC^, and the solution was dialyzed in a 3.5 kDa MWCO Slide-A-Lyzer dialysis cassette (ThermoFisher Scientific) against Buffer C (50 mM Tris, pH 7.5; 200 mM NaCl) at 4 °C overnight. TEV protease was then separated from TagA^ΔC^ by binding 10 mL of suspended His-Pure Co^2+^ resin (Life Technologies) that was pre-equilibrated with Buffer C; cleaved TagA^ΔC^, which lacked the His_6_-tag, was eluted from the resin using Buffer C. Cleaved TagA^ΔC^ was further purified by gel filtration chromatography using a Sephacryl size-exclusion column (GE Healthcare Life Sciences) that was equilibrated with Buffer C. Purified TagA^ΔC^ was then pooled, concentrated to 55 mg/mL, and stored at 4°C. Analytical SEC experiments were performed using a Superdex 75 10/300 Increase column (GE) using Buffer D (50mM Tris, pH 7.5; 500 mM NaCl).

Selenomethionine (SeMet) labeled protein was prepared with cultures grown in M9 minimal media in the presence of kanamycin at 37°C until an OD_600_ of 0.6-0.8 was reached. Protein expression was then initiated by adding IPTG to 1 mM followed by overnight protein expression at 18°C. Protein purification was performed as described for native protein.

**Structure determination**. Recombinant selenomethionine (SeMet) labeled TagA^ΔC^ at a concentration of 50 mg/mL in Buffer C was used for crystal screening. Screening was performed with the JCSG+ broad matrix suite (Molecular Dimensions) at room temperature in a sitting-drop vapor diffusion format (200 nL drop size). SeMet-labeled protein crystals grew over the course of three days in the presence of 200 mM lithium sulfate; 100 mM phosphate citrate, pH 4.2; and 20% PEG 1000. For X-ray data collection, TagA^ΔC^ crystals were cryoprotected using reservoir solution containing 35% glycerol. Diffraction datasets were collected at the Advanced Photon Source (APS) beamline 24-1D-C equipped with a Pilatus-6M detector. All selenomethionine (SeMet) labeled data were collected at 100 K. Data were collected at the detector distance of 300 mm, with 0.25° oscillations, and at a 0.9791 Å wavelength. Multi-wavelength anomalous dispersion (MAD) experiment was collected at peak (12663.0 eV), inflection (12660.3 eV), and high remote (12763.0 eV) energy wavelengths.

The SeMet-TagA^ΔC^ crystals diffracted X-rays to 2.0 Å resolution. The XDS/XSCALE package was used to index, integrate and scale data in P2_1_ space group [1]. The asymmetric unit of the crystal contained eight protein molecules, yielding a Matthews coefficient of 2.11 Å/Da and a 41.79% solvent content in the unit cell. The SHELX suite was used to locate the heavy atom substructure, which identified a total of 56 selenium atom sites [2]. The quality of the phases calculated with the peak, inflection, and high remote energy diffraction datasets were improved using SHARP and the wARP suite (Global Phasing Limited). The heavy atom parameters were refined with MLPhare using the CCP4i suite [3]. Density modification and non-crystallographic symmetry averaging was performed with the CCP4i suite to improve the quality of the electron density map. Automated model building was performed with BUCCANEER, followed by refinement with BUSTER [4]. Modeling of the additional electron density was confirmed using 2F_o_-F_c_ omit maps generated using BUSTER. Complete refinement and structure statistics are reported in **Table 1** (5WB4).

A second crystal form was produced with recombinant TagA^ΔC^ in the presence of UDP and ManNAc ligands. TagA^ΔC^ at a concentration of 45 mg/mL in Buffer C with 10 mM UDP and 10 mM ManNAc was used for crystal screening with the JCSG+ broad matrix suite (Molecular Dimensions) as described above. TagA^ΔC^-ligand co-crystals grew over the course of two days in the presence of 200 mM calcium acetate; 100 mM sodium cacodylate, pH 6.5; and 40% PEG 300. A single wavelength diffraction dataset for a non-cryoprotected crystal was collected at the APS beamline 24-1D-C equipped with a Pilatus-6M detector as described above. All UDP:TagA^ΔC^ crystallography data were collected at 100K. Data were collected with a 600 mm detector distance, with 0.5° oscillations, and at a 0.9791 Å wavelength. The crystals diffracted X-rays to 2.9 Å resolution. The XDS/XSCALE package was used to index, integrate and scale data in P2_1_ space group [1]. The asymmetric unit of the crystal contained six protein molecules, yielding a Matthews coefficient of 2.22 Å/Da and a 44.66% solvent content in the crystal unit cell. The PHASER program in the CCP4i suite was used for molecular replacement, employing the coordinates of the apo-TagA^ΔC^ (5WB4) [5]. Molecular replacement yielded a single solution, which was refined in iterative runs using Buster software. Additional electron density resembling the UDP ligand was observed using 2F_o_-F_c_ omit maps generated by BUSTER [4]. Complete refinement and structure statistics are reported in **Table 1** (5WFG).

TagA carboxyl terminal structure was modeled using Generative Regularized Models of Proteins (GREMLIN), accessed online at <http://gremlin.bakerlab.org>. TagA structure could not be modeled as a dimer in its full length (Dr. Sergey Ovchinnikov, personal communication). Chain A of TagA^ΔC^ and TagA^GM^ were aligned with an RMSD of 2.2 Å.

**Oligomeric Analysis.** The dissociation constant and oligomeric determination of apo- and UDP-bound TagA^ΔC^ were determined by equilibrium sedimentation analytical ultracentrifugation (AUC) on an Optima XL-A analytical ultracentrifuge (Beckman Coulter, Brea, CA). Data regression analysis was performed using the Beckman-Coulter Optima Analytical Ultracentrifuge Origin Data Analysis Package. The data were fit to multi-exponential models and represented best by a monomer-dimer equilibrium that was calculated using the predicted monomeric molecular weight of 21633 Da by the ExPASy ProtParam tool. The dissociation constant (*K*_d_) was determined to be the inverse of *K*_a(conc)_ using equation 1 [6]:

$K_{a\left( \mathrm{conc} \right)} =K_{a\left( \mathrm{abs} \right)} \frac{\left( \varepsilon l \right)^{n-1}}{n}$ (1)

The molar extinction coefficient (*ε*) was determined to be 12950 cm^-1^M^-1^ using the ExPASy ProtParam tool, where *l* is the path length of 1.2 cm, *n* is the order of oligomerization, and *K*_a(abs)_ is the absorbance association constant, as determined by the nonlinear regression of the monomer-dimer multiexponential model using the analytical software program mentioned above.

Chemical crosslinking experiments with disuccinyl suberate (DSS) (ThermoFisher Scientific) was performed according to manufacturer guidelines, with the following variations. RC21tagAFL and RC21tagAG195 overnight cultures were diluted 1:100 into LB, grown at 37°C and induced with 1mM IPTG at OD_600_ = 0.4. After two hours, cells were collected, washed three times with buffer (PBS, pH 8.0) and finally re-suspended at 3X concentration. Ten microliters of sample were loaded onto an SDS-PAGE gel, and western blotting was performed as described above.

***Bacillus subtilis* cloning.** Antibiotic concentrations used in this study, unless otherwise indicated, were 100 µg/mL ampicillin, 1 µg/mL erythromycin, and 100 µg/mL spectinomycin. The full-length *B. subtilis* *tagA* gene was amplified from purified genomic DNA from *B. subtilis* 168 (*Bacillus* genetic stock center) and sub-cloned into the pBL113 shuttle vector (**Table S1**) using *E. coli* XL10 (New England BioLab) to create pHisTagAFL. The hexahistidine-tag was incorporated during Gibson assembly [7]. The truncated TagA construct (residues Met1-Val196) was constructed by amplifying the first 588 nucleotides of *tagA* and cloning into the pBL113 shuttle vector to create plasmid pHisTagAV196. *B.* *subtilis* was made competent as previously reported and transformed with 5-10 µL of pure plasmid to create strains RC168tagAFL and RC168tagAV196 [8]. Homologous double-crossover at the *thrC* locus was verified using tryptophan/threonine auxotrophy and sequencing (Laragen Sequencing). The *tagA* gene was removed via allelic replacement using plasmid p*tagA::spec*, which was constructed by cloning 1 kb of DNA upstream and downstream of the *tagA* gene to flank the spectinomycin resistance cassette in the pIC56 plasmid; a portion of the spectinomycin cassette that was predicted to form a stem loop was removed to ensure that the native operon was not disrupted. Strain RC168tagAFL was made competent as previously described, transformed with an approximately 3 kb linear PCR product from p*tagA::spec*, and plated on LB agar plates with spectinomycin at 37°C to produce strain RC168tagAFLΔtagA. The *tagA* knockout was determined via colony PCR, and the DNA sequence was confirmed (Laragen Sequencing).

**SUPPLEMENTAL REFERENCES**

1. Kabsch W. XDS. Acta Crystallographica Section D. 2010;66(2):125-32. doi: doi:10.1107/S0907444909047337.

2. Thorn A. Experimental Phasing: Substructure Solution and Density Modification as Implemented in SHELX. In: Wlodawer A, Dauter Z, Jaskolski M, editors. Protein Crystallography: Methods and Protocols. New York, NY: Springer New York; 2017. p. 357-76.

3. Winn MD, Ballard CC, Cowtan KD, Dodson EJ, Emsley P, Evans PR, et al. Overview of the CCP4 suite and current developments. Acta Crystallographica Section D. 2011;67(4):235-42. doi: doi:10.1107/S0907444910045749.

4. Bricogne G. Direct phase determination by entropy maximization and likelihood ranking: status report and perspectives. Acta Crystallogr D Biol Crystallogr. 1993;49(Pt 1):37-60. Epub 1993/01/01. doi: 10.1107/s0907444992010400. PubMed PMID: 15299544.

5. McCoy AJ, Grosse-Kunstleve RW, Adams PD, Winn MD, Storoni LC, Read RJ. Phaser crystallographic software. Journal of Applied Crystallography. 2007;40(Pt 4):658-74. doi: 10.1107/S0021889807021206. PubMed PMID: PMC2483472.

6. McRorie DK, Voelker PJ. Self‐associating systems in the analytical ultracentrifuge. Beckman Instruments, Fullerton, CA. 1993.

7. Gibson DG, Young L, Chuang R-Y, Venter JC, Hutchison Iii CA, Smith HO. Enzymatic assembly of DNA molecules up to several hundred kilobases. Nature Methods. 2009;6:343. doi: 10.1038/nmeth.1318.

8. Anagnostopoulos C, Spizizen J. REQUIREMENTS FOR TRANSFORMATION IN &lt;em&gt;BACILLUS SUBTILIS&lt;/em&gt. Journal of Bacteriology. 1961;81(5):741.

9. Sievers F, Wilm A, Dineen D, Gibson TJ, Karplus K, Li W, et al. Fast, scalable generation of high‐quality protein multiple sequence alignments using Clustal Omega. Molecular Systems Biology. 2011;7(1).

10. Arbing MA, Chan S, Harris L, Kuo E, Zhou TT, Ahn CJ, et al. Heterologous Expression of Mycobacterial Esx Complexes in Escherichia coli for Structural Studies Is Facilitated by the Use of Maltose Binding Protein Fusions. PLOS ONE. 2013;8(11):e81753. doi: 10.1371/journal.pone.0081753.

11. Huang GL, Gosschalk JE, Kim YS, Ogorzalek Loo RR, Clubb RT. Stabilizing displayed proteins on vegetative Bacillus subtilis cells. Applied Microbiology and Biotechnology. 2018. doi: 10.1007/s00253-018-9062-x.

12. Steinmetz M, Richter R. Plasmids designed to alter the antibiotic resistance expressed by insertion mutations in Bacillus subtilis, through in vivo recombination. Gene. 1994;142(1):79-83. doi: https://doi.org/10.1016/0378-1119(94)90358-1.
